# Evaluating the in-hospital initiation of 6-monthly paliperidone palmitate: a retrospective study

**DOI:** 10.1192/j.eurpsy.2025.621

**Published:** 2025-08-26

**Authors:** J. Mesones, A. Miller, A. Martínez, G. Carretero

**Affiliations:** 1 Psychiatry, Torrevieja University Hospital, Torrevieja, Spain

## Abstract

**Introduction:**

Long-acting injectable antipsychotic treatments (LAIA) have marked a significant therapeutic advancement. For certain patients, clinical necessity may dictate that the most suitable treatment option is to initiate a 6-monthly LAIA earlier than standard protocols recommend. This can involve transitioning directly from an oral antipsychotic regimen or accelerating the switch from a monthly LAIA, a process known as alternative initiation.

**Objectives:**

The objective of this study is to describe our experience with alternative initiation protocols for 6-monthly paliperidone palmitate (PP3M) in psychotic patients, as implemented at Torrevieja University Hospital.

**Methods:**

This retrospective descriptive study utilized a sample selected through non-probabilistic consecutive sampling over a 27-month period (n=43). The study included patients who initiated 6-monthly paliperidone palmitate treatment through an alternative approach between May 2022 and August 2024 at Torrevieja University Hospital. All alternative initiations were performed in acute patients, who were decompensated at the time of initiating PP6M treatment, with very low therapeutic adherence. A descriptive analysis was conducted, with mean and standard deviation calculated for quantitative variables, and frequencies (N) and percentages calculated for categorical variables.

**Results:**

Over the 27-month duration of this study, PP6M was administered to 43 patients (n=43). The average age was 41 years (SD ± 14.07). 53.49% (n=23) were male and 46.51% (n=20) female. The mean duration of disease progression was 6.67 years (SD ± 7.16). 20.9 % (n=9) of the patients were using substances with abuse potential. The diagnoses were distributed as follows: schizophrenia (32.56%), unspecified psychosis (30.23%), bipolar disorder (11.63%), delusional disorder (11.63%), borderline personality disorder (9.30%), and schizoaffective disorder (4.65%).

A total of 24 alternative initiations were made from oral antipsychotics, 15 from monthly paliperidone palmitate (PP1M), and 4 from aripiprazole LAIA 400 mg/month. During the 27-month study period, there were 4 hospital admissions and 7 visits to the emergency department.

Treatment adherence after the alternative initiation of PP3M was 72 %, with patients completing all administrations on the scheduled dates. 74% of the patients are in outpatient follow-up. One patient experienced extrapyramidal symptoms as a side effect..

**Conclusions:**

The present study indicates that there is a subset of patients who may benefit from an alternative initiation with PP6M, potentially enhancing adherence due to the simplified administration regimen. Initiating PP6M as an alternative, whether transitioning from oral antipsychotics, from PP1M or from another LAIA, could be an effective approach for managing decompensated patients with very low therapeutic adherence, demonstrating excellent tolerability and improved adherence.

**Disclosure of Interest:**

None Declared

